# Cost Effectiveness of Mobile Health for Antenatal Care and Facility Births in Nigeria

**DOI:** 10.29024/aogh.2364

**Published:** 2018-11-05

**Authors:** Diana M. Bowser, Donald S. Shepard, Allyala Nandakumar, Adeyemi Okunogbe, Tyler Morrill, Yara Halasa-Rappel, Monica Jordan, Farida Mushi, Carolyn Boyce, Erhunmwunse Oluwayemisi

**Affiliations:** 1 Heller School for Social Policy and Management, Brandeis University, Waltham, MA, US; 2 Pardee RAND Graduate School, Santa Monica, CA, US; 3 Abt Associates, Cambridge, MA, US; 4 IntraHealth International, Windhoek, NA; 5 Pathfinder International, US

## Abstract

**Background:**

The use of mobile technology in the health sector, often referred to as mHealth, is an innovation that is being used in countries to improve health outcomes and increase and improve both the demand and supply of health care services. This study assesses the actual cost-effectiveness of initiating and implementing the use of the mHealth as a supply side job aid for antenatal care. The study also estimates the cost-effectiveness ratio if mHealth was also used to encourage and track women through facility delivery.

**Methods:**

The methodology utilized a retrospective, micro-costing technique to extract costing data from health facilities and administrative offices to estimate the costs of implementing the mHealth antenatal care program and estimate the cost of facility delivery for those that used the antenatal care services in the year 2014. Five different costing tools were developed to assist in the costing analysis.

**Findings:**

The results show that the provision of tetanus toxoid vaccination and malaria prophylaxis during pregnancy and improved labor and delivery during facility delivery contributed the most to mortality reductions for women, neonates and stillbirths in mHealth facilities versus non-mHealth facilities. The cost-effectiveness ratio of this program for antenatal care and no demand-side generation for facility delivery is US$13,739 per life saved. The cost-effectiveness ratio adding in an additional demand-side generation for facility births reduces to US$9,806 per life saved.

**Conclusion:**

These results show that mHealth programs are inexpensive and save a number of lives for the dollar investment and could save additional lives and funds if women were also encouraged to seek facility delivery.

## Introduction

In the effort to improve maternal health, the global community has embarked on programs to increase demand for antenatal services, increase institutional delivery, identify risks in pregnancy and effectively manage obstetric emergencies. Despite government efforts, Nigeria accounts for approximately 14% of pregnancy related deaths globally [1]. These consistently high rates are a result of poor quality antenatal care, lack of access to antenatal care, and underutilization of care, among other causes [2]. Hence there is a great need to improve antenatal care and increase access and demand for services as well as a better connection to facility births. Against this backdrop, new mobile technologies are being employed to support interventions tackling maternal and neonatal mortality, especially in low- and middle-income countries (LMICs).

The World Health Organization defines mHealth as “the use of mobile and wireless technologies to support the achievement of health objectives [3].” While there is a great deal of optimism about the potential of mHealth for improving health outcomes in LMICs, the evidence base currently is comprised of many smaller scale mHealth initiatives [4,5]. Larger-scale applications coordinated with rigorous evaluations of the impact of mHealth on health system and health outcomes are needed [4,6]. Labrique et al. (2013) has developed a framework for understanding and measuring how mHealth interventions can be integrated into health systems in order to improve some of the main functional areas of health systems [7].

Although many of the evaluations of mHealth programs have been on smaller, pilot studies, they have shown improvement in some broader health system areas from mHealth programs. A systematic review of controlled trials of mHealth interventions by Free et al. found consistent modest benefits associated with mHealth interventions related to clinical support, as well as utilization of health services via appointment reminders [8]. A review by Piette et al. documented benefits in patient self-care and chronic disease management in LMICs derived from mHealth [9]. A preliminary study from Great Lakes Kisumu University found short messaging service (SMS) was more effective than pamphlets in improving knowledge, attitude, and practices of mothers in an LMIC [10]. There is less evidence demonstrating how improving service delivery can impact health outcomes. Lester et al. (2010) demonstrated, using a randomized controlled design, that SMS messaged delivered to patients taking antiretroviral therapy increased their adherence and subsequently decreased their viral loads [11]. Furthermore, Lee at al. conclude that infant feeding can be improved moderately by intervention delivered through SMS messaging [12].

Zeroing in on mHealth interventions in maternal, newborn, and child health (MNCH), parallel themes emerge. Nurmatov et al. note that many of the studies on the impact of mHealth for MNCH tend to be descriptive and aspirational instead of focusing on outcomes and effectiveness [13]. In a literature review and project map on mHealth for MNCH (2013), William Philbrick found a general evidence gap, which he broke down into four gap categories: lack of rigor^1^ in study design, an imbalance in the type of MNCH interventions that have been studied most, the use of non-health outcome measurement indicators, and a lack of cross-cutting approaches that examine the effects of mHealth for MNCH from the perspective of health systems strengthening, scaling up, and reducing inequalities. Nonetheless, Philbrick notes that these gaps are rapidly closing with the volume and rigor of studies increasing over time and the existence of a number of rigorous studies currently underway [14]. In a 2011 literature review, Tamrat and Kachnowski also noted a lack of rigor in study design among the 34 articles on mHealth for MNCH. These articles did indicate positive gains in the areas of MNCH medical emergencies, point of care support, health promotion, and data collection and management [15,16]. Importantly, they also noted a lack of studies that frame mHealth for MNCH in terms of cost-effectiveness, which is important for bringing these interventions to scale [16]. A review by Noordam et al. echoes the themes of lack of rigor, inconsistency, and lack of evidence for scaling up mHealth for MNCH interventions [17]. This last point is important, as mHealth for MNCH will remain pigeonholed in the form of hundreds of small-scale pilot projects, forestalling more widespread impacts if cost-effectiveness studies are not conducted.

Due to the dearth of studies and evidence related to the cost-effectiveness of mHealth interventions, it was necessary to reach beyond mobile-based interventions in the literature for examples of studies examining the cost-effectiveness of MNCH interventions with results quantified as health outcomes. [22] examined the impact of a voucher scheme on maternal mortality in Uganda [22]. Using the Lives Saved Tool (LiST), the authors found the voucher scheme had a cost of $302 per disability-adjusted-life year (DALY) averted, and $20,756 per death averted. The study also found a 52.3% increase in demand for births at health facilities. A systematic review published in July 2014 analyzed 48 published studies and found that both demand and supply side MNCH interventions can be cost-effective, though there are few high-quality studies in this area and many studies use different effectiveness measures [19]. Other more recent studies that include cost analyses are either not focused exclusively on maternal and child health [20] or provide guidelines rather than a rigorous cost effectiveness analysis [21]. Our study seeks to address this gap in the literature by examining the cost-effectiveness of an mHealth intervention in two regions of Nigeria.

## Data and Methods

A matched case control cohort study design was used to conduct a cost-effectiveness analysis in 20 health facilities in Nigeria (10 cases and 10 controls) to estimate the incremental costs of providing antenatal care services using a mobile device (CommCare^2^ mHealth device) for case management and decision support. Ten health facilities that received funding and technical support from Pathfinder International to implement the mHealth program were chosen as “cases”. Five of the cases were from the state of Abuja, and five of the cases were from the state of Nassarawa. Ten additional facilities were then chosen as matched controls to test the counterfactual of what happened in facilities that did not receive funding and technical support from Pathfinder International to implement the mHealth program. Five of the matched facilities were from Abuja and five were from Nassarawa.

The 10 controls were selected using several criteria to ensure that the controls matched as closely as possible key characteristics of the mHealth sites. The controls were matched in the following criteria: had to offer ANC care, should be giving Iron-Folic Acid supplementation during ANC, had IPT for malaria prevention, and were offering anti-malarial treatment. The average catchment areas as well as the average ANC attendees per month for the cases and controls were chosen to be as close as possible to the cases. The criteria used were summarized in Table 1 below.

**Table 1:** Indicators used for matching the controls with the case facilities.

	Offers ANC June 2012	Gives IFA June 2012	Catchment Areas 2014	Average ANC attendees/month June 2012	Has IPT for prevention 2012	Anti-malarial treatment 2012

**Abuja Cases**						

Pyakasa	Yes	Yes	12,419	20	Yes	Yes
Gbagalpe	Yes	Yes	19,967	100	No	No
Ketti	Yes	Yes	36,824	20	No	Yes
Kobi	Yes	Yes	16,290	6	No	No
Idu Karimi	Yes	Yes	6,972	11	No	No
Total			92,472	157		
**Abuja Controls**						

Bassan’jiwa	Yes	Yes	81,000	6	Yes	Yes
Dutse Garki	Yes	Yes	1,488	30	Yes	Yes
Jahi PHC	Yes	Yes	1,725	12	Yes	Yes
Kabusa PHC	Yes	Yes	3,050	19	Yes	Yes
DamangorovPHC	Yes	Yes	17,525	56	Yes	Yes
Total			104,788	123		
**Nassarawa Cases**						

Keffi Wambai	Yes	Yes	4,700	5	No	Yes
Lafia East	Yes	Yes	17,350	180	No	Yes
Mana PHC	Yes	Yes	14,360	80	Yes	Yes
Sabon Gari	Yes	Yes	8,089	24	Yes	Yes
Nassarawa Egon	Yes	Yes	34,065	60	Yes	Yes
Total			78,564	349		
**Nassarawa Controls**						

Adogi	Yes	Yes	26,175	127	No	Yes
Angwon Toni	Yes	Yes	6,502		No	Yes
Akuba Lafia	Yes	Yes	10,972	85	Yes	Yes
Agyaragu	Yes	Yes	6,502	81	Yes	Yes
Tudan Gwandara	Yes	Yes	13,500	82	Yes	Yes
Total			63,651	375		

## Data Collection

### Cost Data

Four different costing tools were developed and then used to collect the costing data in each of the 20 mHealth facilities for the year 2014, one year after implementation of the mHealth job aid. Each of these tools are described below.

*Facility Capital and Recurrent Costing Tool*: The facility costing tool was used to collect data on capital costs (e.g. buildings, equipment, vehicles) and recurrent costs, including non-medical consumables (e.g. water and electricity) and medical consumables (e.g. drugs, test kits, vaccinations) for each facility over the period January to June 2014. The value of all donated goods and services including medical equipment, drugs, and personnel and their use for services related to the mHealth device were also included in the analysis.

Capital costs included all capital items purchased specifically to be used by the project in each of the health facilities. The useful life for each product was extracted from guidelines for recovery periods for different property classes according to the US government Internal Revenue Service (IRS, 2014). The useful life for project cell phones and tablets was estimated at three years – the life of the project. Costs for all capital items were provided by the Pathfinder Nigeria country team.

All recurrent costs were estimated for the year 2014 using data on the amount of drugs (Folic Acid, Iron, Multivitamin, Vitamin B Complex, Fansidar, ACT Antimalarial Coartem, Paracetamol, Tetanus Toxoid), laboratory products (Hepatitis C, Hepatitis B, RVS (HIV), VDRL (Syphilis), Malaria, Blood Group RhD, Urine Analysis, and Anemia (PCV)), and supplies and services purchased (gasoline, vehicle maintenance or repair, utilities, office supplies, rent, travel) by each of the 10 facilities over each of these years. The total amount of these costs was calculated using one of the following six different costing methods depending on the data available and collected through the Facility Costing Tool: 1) the product of the unit cost for each drug and/or laboratory by number of tablets given to each woman per month by the average number of first antenatal care visits per month by 12 months in a year; 2) same as method one only average unit cost from other facilities was used for those facilities which did not have recorded unit costs; 3) the product of unit cost multiplied by number of tablets/dose per woman for the entire course of her pregnancy by the total number of women that came in for a first antenatal care visit; 4) the product of yearly total cost by the percent of the of the product that was reported to be used for the antenatal care services by the percent of the total antenatal care population that came in for their first antenatal care visit; 5) the product of unit cost by the total number of women that come in for a first antenatal care visit; and 6) the total cost multiplied by the percent of total facility registration that comes in for a first antenatal care visit.

*Facility Personnel Costing Tool*: The facility personnel costing tool was used to collect data on clinical and non-clinical personnel and their salary levels. Salaries for each type of personnel were extracted from the Local Government Unit in Abuja, Nigeria. The percent of effort attributable to antenatal care services, for those personnel who worked on antenatal care and other services, was calculated based on the number of women that came to the facility for their first antenatal care visit divided by the entire registered patients in the facility.

*CommCare Costing Tool*: The CommCare Costing tool, developed by the Research Team, was used to collect costs associated with setting up the CommCare device on mobile phones and tablets in Nigeria. The costs included in this analysis were activities related to developing the software with the firm Dimagi Inc. and rolling out the mHealth program in Nigeria. Two costing scenarios were utilized in the analysis. The mHealth costs incorporated training for staff in country as well as the consulting fees for Dimagi (amortized for three years), cell phones and tablets (amortized for two years), and relevant recurrent costs for years 2012, 2013, and 2014 (participant support, purchased services, supplies and equipment, and travel).

*Societal Costing Tool*: A societal costing tool was developed to measure the direct and indirect costs by the women when they come to the facility for their ANC visits. This tool was administered to a sequential sample of 10–15 women from five randomly selected mHealth study facilities; three in Abuja and two in Nassarwa. The women were asked to voluntarily participate in societal interview upon leaving the antenatal clinic. The interview asked the women to provide estimates of the additional direct or opportunity costs of seeking care for themselves or those who accompanied them to the facility including transportation fees, the cost of seeking professional care (in the case of a referral), time lost from work or school to seek care, as well as time lost from work, school, or leisure by those that accompanied them to the facilities.

Societal costs were estimated by calculating the direct non-medical costs included patients’ out-of-pocket expenditures on transportation and food associated with obtaining an antenatal visit at a health center. The indirect costs were the monetary value of days of school lost and income lost due to the antenatal visit incurred by patients and their caregivers. The cost of a school days lost was obtained from several relevant sources in Nigeria that provided information on the number of public school days as well as the cost of specific items such as text books, uniforms, and other text book materials. The cost of a school day lost is the product of the daily cost of a public school day and the average school days lost. The economic cost attributed to an antenatal care visit was computed as summation of the average direct non-medical and indirect costs.

#### Other Data Extracted and Used in the Analysis

Additional cost data were extracted from published literature. For example, the costs for facility births were extracted from published literature on facility costs in similar countries as well as communication with staff in health facilities in Nigeria. The estimated costs for a delivery were the following: US$19.42 (public health facility), US$76.36 (private health facility), US$43.50 (general government hospital). These costing numbers were generated based on in country interviews regarding costs of public and private delivery and published costing data from sub-Saharan Africa [22]. Each of these costs were applied to the total number of new ANC cases in each facility and the estimated number of these ANC cases that would deliver in each type of facility.

The National Health Management Information System (NHMIS), Nigeria data were used to verify the total number of new ANC cases per facility in the year 2014. The NHMIS data were also used to verify where each of these ANC cases, calculated over a one-year period, delivered. More specifically, the percent of total deliveries that were classified as “spontaneous vaginal deliveries” or classified as “Cesarean sections, complications, preterm delivery, or referral” were used to calculate the percent of public, general hospital deliveries and the percent of general hospital deliveries, respectively. Using the ratio of 1.7 hospital deliveries and health facility deliveries in comparison to private and assisted at-home deliveries according to the 2014 DHS (24.5% versus 14.4%), the total percent of private and assisted at-home deliveries was calculated for each facility. DHS data also showed that assisted at home deliveries were 27% of private and assisted deliveries at home. This number was used to calculate the percent of assisted deliveries at home. The remainder of deliveries were private facilities and unassisted. The percentages of types of deliveries for each facility were applied to the total first ANC visits to calculate how many types of deliveries for each facility and the cost for each type of delivery.

### Health Facility Data

NHMIS data were extracted to analyze patterns of utilization with respect to key ANC indicators for both cases and controls in Abuja and Nassarawa. Table 2 shows the data that were extracted from the NHMIS databases as well as the dates of extraction.

**Table 2:** Data extracted from NHMIS database.

Variable Name	Extraction Dates

ANC proteinuria test done	2014, monthly
ANC proteinuria test positive	2014, monthly
ANC syphilis case treated	2014, monthly
ANC syphilis test done	2014, monthly
ANC syphilis test positive	2014, monthly
Antenatal attendance – Total	2013–2015, monthly
Antenatal first visit – Total	2013–2015, monthly
Deliveries – Complications	2013–2015, monthly
Deliveries – Preterm	2013–2015, monthly
Deliveries – Caesarian Section	2013–2015, monthly
Deliveries – SVD (Spontaneous Vaginal Delivery)	2013–2015, monthly
Deliveries taken by a skilled birth attendant	2013–2015, monthly
Deliveries – Total	2013–2015, monthly
Delivery Assisted	2013–2015, monthly
Facility attendance – Total	2013–2015, monthly
OPD attendance – Total	2014, monthly
Persons with clinically diagnosed malaria	2014, monthly
Pregnant women who received Haematinics’ IFAs-Iron and Folic Acid supplements	2014, monthly
Pregnant women who received malaria IPT1	2014, monthly
Pregnant women who received malaria IPT2	2014, monthly
TT1 for nonpregnant women	2014, monthly
TT1 for pregnant women	2014, monthly
TT2 for nonpregnant women	2014, monthly
TT2 for pregnant women	2014, monthly
Women referred out for pregnancy related complications	2013–2015, monthly

NHMIS data above were used to calculate coverage of antenatal care and delivery in case and control facilities in the year 2014. Four different levels of facility births were calculated using the NHMIS data: public facility delivery, private facility delivery, general hospital delivery, and unassisted delivery. Private deliveries and unassisted delivery rates were calculated using both NHMIS data and ratios of specific types of delivery from the DHS 2014.

Mortality rates were extracted from published literature as follows: maternal mortality rate of 392/100,000 live births [23], stillbirth mortality rate of 42/1,000 live births [24], and neonatal mortality rate of 35/1,000 live births [25] for North Central Nigeria (Table 3).

**Table 3:** Data Description for Antenatal Care and Facility Delivery Coverage Indicators.

Antenatal Care Indicators	

Tetanus Toxoid Coverage (%)	Number Tetanus Toxoid 1 given/Number of ANC 1 patients
Malaria IPT Coverage (%)	Number Malaria IPT 1 given/Number of ANC 1 patients
Iron/Folic Acid (IFA) Coverage (%)	Number IFA given to ANC/All ANC
Hypertension Screening^3^ Coverage (%)	Number ANC proteinuria test done/All ANC
Syphilis Detection^4^ Coverage (%)	Number ANC syphilis test done/All ANC
**Facility Delivery Indicators**	

Public Facility Delivery (%)	Number of spontaneous vaginal deliveries recorded in the health facility/All ANC
Private Facility Delivery (%)	Percent of private facility deliveries*
General Health Facility (%)	Number of Caesarean sections, complications, preterm deliveries and referrals/All ANC
Unassisted Deliveries (%)	Percent of Unassisted facility deliveries**

Source: DHIS2 Health Management Information System; *Calculated using NHMIS data and DHS ratio of private to public deliveries, **Calculated using NHMIS data and DHS ratio of unassisted to private deliveries.

Table 4 reports the catchment populations for each of the 20 facilities included in the analysis as this provided the demographic cohort that was used to calculate lives saved for specific cohorts. The catchment areas were calculated based on reported numbers from facilities, reported numbers from the Local Government Authority (LGA), general population size per facility, and population size based on number of pregnant women visiting the facility.

**Table 4:** Reported and Calculated Catchment Areas for the 20 Facilities.

Catchment Area Cases and Controls			

Cases	2014	Controls	2014

Pyakasa	12,419	Bassan’jiwa	81,000
Gbagalpe	19,967	Dutse-Garki	1,488
Ketti	36,824	Jahi	1,725
Kobi	16,290	Kabusa	3,050
Idu Karimi	6,972	Gidanmangoro	17,525
**Total Abuja**	**92,472**	**Total Abuja**	**104,788**
Keffi Wambai	4,700	Agyaragu	6,502
Lafia East	17,350	Adogi	26,175
Mana PHC	14,360	Akuba Lafia	10,972
Sabon Gari	8,089	Angwon Toni	6,502
Nassarawa Egon	34,065	Tudan Gwandara	13,500
**Total Nassarawa**	**78,564**	**Total Nassarawa**	**63,651**
**Total**	**171,036**	**Total**	**168,439**

## Data Collection and Maintenance

All data collected were maintained completely confidentially. Any financial data related to salaries and other benefits were linked with title of health staff only (physicians, nurse, etc.) and not identified by individual names. All data were cleaned and analyzed by the analytic team. Data were transferred from the data collection tools to Excel and Stata for analysis.

## Data Analysis

The LiST Tool Spectrum Software was used to calculate the impact of the mHealth program by comparing coverage between cases and controls for the five antenatal care indicators and the facility delivery indicators. The LiST Tool used effectiveness and affected fractions to estimate the lives saved from implementing the level of service based on the coverage indicators for cases and controls. Using the catchment areas of the 20 facilities, lives saved for women and children were calculated based on the increased coverage of five specific interventions offered during ANC and the additional facility births. The LiST Tool used an equation to estimate the mortality reduction and then lives saved specific cause of death due to specified interventions. The calculation was used to estimate the impact of the five different ANC interventions and additional facility births on maternal mortality, stillbirth, and deaths for children under one year of age.

Two scenarios were estimated for the analysis below. First, the additional lives saved from the implemented ANC activities were calculated for all cases and controls. As part of this first scenario, the additional lives saved from facility delivery using the reported place of delivery was calculated for cases and controls. The second scenario maintained the ANC coverage indicators levels in cases and controls, but also estimated how many additional lives would be saved from facility delivery if the number of unassisted deliveries fell to 50% in all mHealth facilities.

## Results

Table 5 summarizes the difference in coverage rates for the five ANC coverage indicators and for the different types of facility delivery for cases and controls in Abuja and Nassarawa. For all five ANC coverage indicators, with only one exception, there is a higher percentage coverage in cases in comparison to controls. The one exception is for hypertension screening, where the controls in Nassarwa have achieved 49% while the mHealth facilities in Nassarawa achieved 27%. Notably, all women reported coming in for their first ANC visit in the Abuja mHealth facilities received their first tetanus toxoid vaccination, their first malaria prophylaxis (IPT1) and iron/folic acid supplementation.

**Table 5:** Difference in Coverage Indicators between Cases and Controls, Abuja and Nassarawa.

Coverage Indicators	Abuja		Nassarawa	

	Case	Control	Case	Control

**ANC**				

Tetanus Toxoid Coverage (%)	100	73	72	39
Malaria IPT Coverage (%)	100	71	77	35
Iron/Folic Acid (IFA) Coverage (%)	100	85	100	100
Syphilis Detection Coverage (%)	31	19	54	0
Hypertension Screening (%)	50	26	27	48
**Deliveries**				

Public Facilities Delivery (%) (Emoc)	8.95	12.73	10.45	13.54
Private Facilities Delivery (%) (Bemoc)	6.38	5.93	5.23	6.06
General Hospital Delivery (%) (Cemoc)	5.93	1.10	1.76	0.60
Unassisted Deliveries (%)	76.37	78.04	80.61	77.55

There is no notable difference in the type of deliveries between cases and controls in Abuja and Nassarawa. There are more public facility deliveries, general hospital deliveries, and unassisted deliveries in the controls versus the cases in both areas. The difference in the use of private facilities for deliveries is not consistent in mHealth versus non-mHealth facilities.

Table 6 shows the reduction in mortality for the 20 health facilities in Abuja and Nassarawa for the five antenatal care interventions and the increased type of facility chosen delivery. In total, providing antenatal care assisted with the mobile hand-held device saved 4.661 additional lives, including women, neonates and stillbirths, in cases versus controls. The most lives saved were from providing tetanus toxoid vaccinations and malaria prophylaxis. Providing improved obstetric care through increased facility delivery after antenatal care can lead to an additional 0.054 lives saved in mHealth facilities.

**Table 6:** Maternal, Stillbirth and Neonatal Deaths Averted from ANC Coverage and Facility Deliveries in Cases versus Controls in Abuja and Nassarawa.

Antenatal Care Interventions	Facility Deliveries: Actual level of unassisted deliveries		Additional Facility Deliveries: Unassisted deliveries 50%

Tetanus Toxoid Coverage	1.116		
Malaria IPT Coverage	1.311		
Hypertension Screening Coverage	0.081	0.054	8.052
Syphilis Detection Coverage	2.146		
Iron/Folic Acid (IFA) Coverage	0.006		
**Total Lives Saved**	**4.661**	**0.054**	**8.052**
**Disability Adjusted Life Years (DALYs)**			

**Total Lives Saved (Antenatal Care and Facility Delivery)**	**4.71**		**12.71**
**Total DALYs**	**109**		**295**

Table 6 also shows the additional lives that could have been saved if the mHealth intervention also encouraged women to attend a health facility rather than having an “unassisted delivery”. Reducing unassisted deliveries to 50% in all mHealth facilities could have saved an additional 8.052 lives.

Table 7 reports the total costs for all 10 health facilities in Abuja and Nassarawa to initiate and implement the mobile hand-held device program for antenatal care over the years 2012, 2013, and 2014. The costs are presented in the traditional cost categories related to personnel costs, recurrent costs, and capital costs. Case facilities have lower personnel costs, perhaps showing some link to efficiency with the mobile device. Cases show higher recurrent and societal costs. Control facilities reported high capital items and capital costs. The results show that there is minimal difference in costs between the case and control facilities.

**Table 7:** Cost for Initiation and Implementation of antenatal care assisted with mobile hand-held device (US$) (Standard Deviation in parentheses).

Cost Categories	Cases	Controls

Personnel	84,388.19 (5,713.83)	95,894.86 (5,964.96)
Recurrent	44,294.37 (5,321.51)	42,957.27 (2,534.05)
Capital	52.48 (6.25)	956.17 (74.70)
Societal	25,049.95 (3,070.28)	20,080.08 (1,404.59)
**Total Costs (US$)**	**153,784.99 (13,555.11)**	**159,888.38 (7,050.17)**
**Average Cost per Facility**	**15,378.49 (1,355.51)**	**15,988.84 (705.02)**

Table 8 reports the total facility delivery costs associated with increased antenatal care for between cases and controls, based on the current facility and unassisted deliveries as well as reducing the unassisted deliveries to 50%. The results in Table 8 show that costs for deliveries do not vary between cases and controls when the mHealth program focuses only on ANC and does not encourage facility delivery. When unassisted deliveries in case facilities are reduced to 50%, the cost for deliveries increases.

**Table 8:** Facility Delivery Costs Associated with Increased Antenatal Care (US$) (Standard Deviation in Parentheses).

Cost Categories	Facility Deliveries: Actual level of unassisted deliveries		Additional Facility Deliveries: Unassisted deliveries 50%	

	Cases	Controls	Cases	Controls

Facility Deliveries	$38,378 (4,365.83)	$34,916 (3,365.17)	$98,240 (10,827.67)	$34,916 (3,365.17)

### Cost Effectiveness Results

Table 9 reports incremental costs between cases (mHealth) and controls (non-mHealth) for two scenarios. The first scenario captures the lives saved for the mHealth program that mainly focused on ANC interventions with minimal demand-side interventions linking women to facility births. As shown in Table 9, the cost-effectiveness ratio for the standalone ANC program is $13,155 per life saved and the cost-effectiveness ratio capturing the minimal number of addition lives saved from facility births (0.054 lives saved) is $13,739 per life saved. The last column of Table 9 captures that additional lives saved if there is a demand-side component that increases the number of unassisted deliveries in the mHealth facilities to 50%. With additional lives saved from facilities deliveries, the cost-effectiveness of the program falls to $9,806. The results show that while it is more expensive to add in facility births, with the additional lives saved the investment is more cost-effective.

**Table 9:** Cost Effectiveness Ratios, Cases versus Controls, 2014.

	Baseline lives saved		Lives saved with increased unassisted deliveries to 50%	

	Cases	Controls	Cases	Controls

**Costs**				

ANC	$153,785	$159,888	$153,784	$159,888
mHealth	$67,407	$0	$67,407	$0
Total	$221,192	$159,888	$221,192	$159,888
Change (ANC)	$61,304		$61,304	
Delivery	$38,378	$34,916	$98,240	$34,916
Change (Delivery)	$3,462		$63,324	
Change (ANC + Delivery)	$64,766		$124,628	
**Effectiveness (ANC only)**					

Lives Saved	4.66		4.66	
DALY Averted	108		108	
**Effectiveness (ANC and facility delivery)**				

Lives Saved (Facility Delivery)	0.054		8.05	
Lives Saved (ANC + Delivery)	4.71		12.71	
DALY (Facility Delivery)	1		186	
DALY (ANC + Delivery)	109		295	
**Cost Effectiveness (ANC only)**				

Per life saved	$13,155		$13,155	
Per DALY averted	$568		$568	
**Cost Effectiveness (ANC + Facility Delivery)**				

Per life saved	$13,739		$9,806	
Per DALY averted	$594		$424	

## Discussion

This is one of the first studies to document costs of implementing a mobile health program to support the delivery of antenatal care services and subsequently increase the number of deliveries in health care facilities. The results show that initiating and implementing a mobile health program for antenatal care is relatively inexpensive and can save lives in comparison to facilities that did not implement the program. We found that implementing the mHealth job aid for antenatal care alone equated to US$13,155 per life saved and US$568 per DALY averted. Including facility birth from a standalone ANC program increased the cost-effectiveness ratios slightly to US$13,739 per life saved and US$594 per DALY averted. However, adding in a simulated demand component that increased unassisted deliveries to 50% in mHealth facilities, reduces the cost-effectiveness to $9,806 per life saved and US$424 per DALY averted.

Due to a dearth of studies examining the cost-effectiveness of mHealth tools to improve antenatal care, there were few studies that provided a direct comparison to the results above. However, the results shown were comparable to similar maternal and child health programs that incorporated antenatal care, facility delivery, or both programs, even if that did not use a mobile device during the implementation of these programs. For example, Colburn et al. (2013) reported a cost effective figure of less than Malawi’s GDP per capita (US$ 5,400) per newborn death averted for a program focused on quality improvement and women’s groups in Malawi [18]. This is lower than our figure for a standalone ANC program (US$13,739), but similar to our estimated figure that reduced unassisted deliveries to 50% ($9,806 per life saved). Another study that examined the cost-effectiveness of a voucher program combined with improved obstetric care in health facilities in Uganda found much higher cost results [22]. They found cost-effectiveness ratios of US$302 per DALY averted and US$20,756 per life saved.

Our results also compare favorably to the willingness to pay figure for Nigeria. The WHO defines programs as cost-effective if they are below the average citizen’s willingness to pay for these services, using the country GDP/capita. The cost per DALY averted through the mobile health program, including both antenatal care and facility births, in Nigeria (US$309/DALY averted) is well below the US$3005 GDP/capita for the year 2014.

The results also demonstrate the more important antenatal care interventions that save the most lives. The methodology utilized focused on five interventions that are commonly offered during antenatal care: tetanus toxoid vaccination, malaria prophylaxis during pregnancy (IPTp), iron folate supplementation, syphilis detection, and monitoring of hypertension during antenatal care. The results demonstrated that the interventions that saved the most lives are tetanus toxoid and malaria prophylaxis. As shown above, some mHealth facilities were achieving 100% coverage of these interventions by the year 2014. Improving the provision of these services had the largest impact on lives saved for both mothers, neonates, and stillbirth. Since there was not a comparable increase in the control facilities, without the mHealth intervention, we can assume that this increase in services was attributed to the mHealth intervention and job aid. This is not surprising as the effectiveness of both these interventions has been shown in the literature [16,26]. The results of this study enforce the idea that better monitoring and compliance of these services can be achieved, in low-income areas that do not have computers or electronic records, with mobile devices. The health care workers reported it was easy to track and monitor the services given to women using the mobile device. The women reported feeling more confident in the services provided by these facilities, especially as they did not have to recall whether they had received the tetanus toxoid vaccination or been previously given the malaria prophylaxis.

The results from this study demonstrate and lend support to other mHealth interventions, specifically around improving the health outcomes of people in LMICs. However, as with other studies, the challenge for program implementers rests on their ability to convince donors that the program will be sustainable. This involves laying out a long-term vision of sustainability beyond the timeframe of donor investment. Addressing the financial gap to ensure that mHealth programs are sustainable remains a daunting task in LMICs settings [27]. A study analyzing the success of the mobile for reproductive health (m4RH) intervention in Tanzania explores three strategies for financial sustainability; revenue generation, cost reduction and strategic partnerships development [28]. The study concludes that a break-even point for financial sustainability can only be achieved when program costs equal revenue generation. Mangone et al. acknowledged a critical gap for service provision to the very poor and underserved in the society, who cannot pay for these services. Going by their strategies, continued reliance on donor funding to provide mHealth services to this population group is inevitable.

There were some limitations in the analysis. There are approximately ten major antenatal care interventions that could potentially save the life of a woman and her baby. Due to data limitations and some policy restrictions in Nigeria, we were not able to track the provision of services related to all ten antenatal care interventions. For this reason, we focused on five major antenatal care interventions reported above. There were also some assumptions made regarding the measure used to estimate provision of each of the five antenatal care interventions. For example, we assumed that each woman who had protein in her urine measured would then be appropriately screened and managed for hypertensive disease during pregnancy with a positive test. We did not have follow-up data to monitor compliance with hypertension management during pregnancy. Similarly, we also assumed that women who received a tetanus toxoid vaccination during their first antenatal care visit would receive a second tetanus vaccination during their second visit. While the health care workers in the facility confirmed this protocol, we did not have the appropriate data to track the subsequent second tetanus toxoid vaccination. We also assumed full compliance regarding the women who were given iron folate and malaria prophylaxis. In future analyses, these supply- and demand-side factors need to be incorporated into the costing analysis [29].

## Conclusion

As the use of mobile devices for many types of health care programs continues to rise, the results from this innovative study will be useful to those planning, implementing, and analyzing the results of these programs. The methodology detailed above can be replicated in several different scenarios. The results are comparable to different programs and provide useful benchmarks to program planners. The results of this study are especially important for governments as they plan, implement, and monitor similar programs in their countries.

## Footnotes

Comparison groups, baseline data, clear and full description of methodology, justification for methodology, theory-based, sufficient sampling size.

CommCare is a free open source mobile platform that enables anyone to build mobile apps. It is utilized across sectors from healthcare to supply and logistics, used mostly by frontline programs as a job aid to reach the last mile.

Note that the Lives Saved Tool refers to this mechanism as Hypertension Screening Coverage.

Note that the Lives Saved Tool includes as part of this indicator treatment, however adherence to treatment was not measured in our study.
